# Tumor-Homing of Mesenchymal Stem Cells Infected with Oncolytic Virus in a Canine Patient

**DOI:** 10.3390/vetsci9060285

**Published:** 2022-06-09

**Authors:** Pablo Delgado-Bonet, Beatriz Davinia Tomeo-Martín, Gustavo Ortiz-Díez, Ana Judith Perisé-Barrios

**Affiliations:** 1 Biomedical Research Unit, Universidad Alfonso X el Sabio, 28691 Villanueva de la Cañada, Spain; pdelgbon@uax.es (P.D.-B.); btomemar@uax.es (B.D.T.-M.); 2 Small Animal Surgery Service, Veterinary Teaching Hospital, Universidad Complutense de Madrid, 28040 Madrid, Spain; gusortiz@ucm.es

**Keywords:** Celyvir, oncolytic virus, tumor-homing, virotherapy

## Abstract

Intravenous administration of oncolytic adenovirus (OAds) can be challenging, although various vehicles for the delivery of the virus to the tumor have been described. The efficacy of mesenchymal stem cells (MSCs) as a virus vehicle has been reported in mouse models and canine and human patients, but the actual action mechanism has never been described in patients. It is of importance to determine whether MSCs infected with OAds can reach the tumor and release the virus in a clinical setting. For this purpose, GFP-labeled MSCs were infected with an OAd and inoculated into a companion dog diagnosed with spontaneous lung carcinoma. Forty-eight hours later, the tumor was excised and analyzed microscopically by flow cytometry for GFP fluorescence detection, and a cellular culture was established. Peripheral blood samples were taken to quantify the oncolytic adenovirus by qRT-PCR. Green fluorescence cells detected in the cellular culture by microscopy and flow cytometry revealed 0.69% GFP-positive cells in the tumor. OAd in peripheral blood was confirmed by qRT-PCR during follow-up. For the first time, the tumoral-homing capacity of OAds infected-MSC has been confirmed in a clinical setting, helping to explain the clinical response mechanism, whose efficacy was previously reported in canine and human patients.

## 1. Introduction

Oncolytic virotherapy is one of the most significant advances in cancer immunotherapy. Virotherapy has shown clinical benefits, leading to EMA (European Medicines Agency) and FDA (United States of America Food and Drug Administration) approval of Imlygic, which is a weakened form of herpes simplex virus 1, for the treatment of human patients with metastatic melanoma [1,2]. Veterinary research with oncolytic virus (OVs) is still not as well developed as in human medicine; however, in the last few years the interest in these therapies is growing [3,4]. Recently, some canine studies using OVs, belonging to the Adenoviridae, Paramyxoviridae, Poxviridae, and Reoviridae families, have been reported [5,6,7,8,9,10]. The OV-treated dogs had mostly solid tumors, such as osteosarcomas, melanomas and mammary carcinomas, or soft tissue sarcomas [4,11].

The administration route of OVs can be challenging, especially for inaccessible tumors or when intratumoral administration is very risky, such as in brain or pulmonary tumors. For those cases, systemic administration is a much safer option. Unfortunately, viral particles (vp) can be retained by the liver or blocked by neutralizing antibodies or other blood components [4,12,13]. Furthermore, the presence of virus in peripheral blood induces the activation of the immune system, leading to an antiviral response. In order to avoid these obstacles, several alternative delivery systems have been proposed recently, including “vehicles” such as cellular carriers, liposomes, nanoparticles and polymeric particles with the ability to transport the virus to the tumor site [14,15,16].

Mesenchymal stem cells (MSCs) exhibit natural tumor tropism, which is the reason why they have been used as cellular carriers to deliver OVs to tumors [15,16,17]. An oncologic immunotherapy agent called Celyvir that can be administered IV and is based on MSCs infected with OAds has been reported [7]. The use of MSCs as vehicles not only reduces the antiviral immune response, but also increases the oncolytic effect of the treatment compared to treatments using the virus without a carrier [15,16,17,18,19].

In the first human clinical trial, Celyvir was used with autologous MSCs to treat relapsed/refractory solid tumors in pediatric and adult patients [18,20]. On the other hand, a veterinary clinical trial report, in which 27 dogs were treated with canine Celyvir (dCelyvir), showed a clinical benefit in 74% of the dogs, including a 14.8% rate of complete remission [7]. Up to now, the tumor-homing capacity of Celyvir has been described in murine models [17,21,22,23] after IV administration. However, the homing capacity of Celyvir has never been described in patients. This study reports the tumor homing capacity of dCelyvir in a canine patient with spontaneous lung carcinoma. To our knowledge, this is the first study involving a patient demonstrating that infected-MSCs can reach tumors.

## 2. Materials and Methods

### 2.1. Study Design

The canine patient was a 10-year-old, 20.3 kg, neutered male mix-breed dog, with a two-month history of a mild cough. Physical examination revealed no significant findings. Three-view thoracic radiography revelated a soft tissue mass in the ventral region of the middle lobe of the right lung. An ultrasound-guided fine-needle aspiration of the mass confirmed a malignant epithelial tumor. An ultrasound examination of the abdomen was normal. Thoracic computerized tomography was also performed with the aim of characterizing the pulmonary mass, and showed the presence of a well-circumscribed oval cavitated mass with smooth margins in the middle lobe of the lung and a moderately enlarged right tracheobronchial lymph node. No other pulmonary nodules were present. Hematology and serum biochemistry results were within normal limits. Based on the diagnosis, lobectomy and excision of the tracheobronchial lymph node was recommended to the owner by the veterinary staff of the Complutense Veterinary Teaching Hospital (Madrid, Spain).

### 2.2. Surgery 

After 48 h of IV dCelyvir infusion, a thoracotomy was performed to remove the middle lobe of the right lung and the enlarged peribronchial lymph node. The surgery was performed at the Complutense Veterinary Teaching Hospital by a board-certificated small animal soft tissue veterinary surgeon, certificated by the Spanish Veterinary Association of Small Animal Specialists (AVEPA). Anesthesia: Methaperformed (0.3 mg/kg; Semfortan, Dechra, Northwich, UK) was administered intramuscularly (IM) as preanesthetic medication. After 25 min, the dog was preoxygenated with 100% oxygen (3 L/min) via face mask for 5 min. Anesthesia was induced by intravenous (IV) administration of propofol (3 mg/kg; Propofol Lipuro 1%, BBraun VetCare, Tuttlingen, Germany) and midazolam (0.2 mg/kg; Midazolam 15 mg/3 mL; Normon, Madrid, Spain). Following intubation, general anesthesia was maintained with isoflurane vaporized in 100% oxygen before moving the patient to the operation room. An ultrasound-guided serratus plane block was performed with bupivacaine (2 mg/kg; Bupivacaine 0.5%, B. Braun, Melsungen, Germany) as a loco-regional anesthetic technique to desensitize the thoracic wall. The neuromuscular blocking agent atracurium (0.2 mg/kg; Atracurium Besylate 10 mg/mL, Inibsa, Barcelona, Spain) was administered IV and mechanical ventilation was instituted. Intraoperative monitoring consisted of continuous electrocardiogram, pulse oximetry, invasive arterial blood pressures, capnography, body temperature and end-tidal isoflurane. Carprofen (4 mg/kg; Rimadyl; Zoetis, Madison, NJ, USA) and cefazolin (20 mg/kg, cefazolin, Normon) were administered perioperatively. Intrapleural bupivacaine (1 mg kg^−1^) was administered at the end of the surgery and every 6 h thereafter for 48 h. Surgery: A right fifth intercostal thoracotomy was performed. No adhesions between the mass and the thoracic wall were identified. A TA stapler (TA30V3L; Covidien, Mansfield, OH, USA) was used to perform the total lobectomy. A gentle blunt dissection was performed to excise the right tracheobronchial lymph node. A chest drain was used for 48 h after surgery. The dog recovered uneventfully and was discharged three days later with 2.2 mg/Kg carprofen (Rimadyl; Zoetis) orally, twice daily for 7 days and clavulanic acid potentiated amoxicillin (Synulox; Zoetis) orally, twice daily for 7 days.

### 2.3. Isolation of dMSCs and Cell Culture 

Adipose tissue was obtained from a healthy donor dog using standard surgical procedure. Adipose tissue samples were homogenized, digested by collagenase B during 45 min at 37 °C, filtered through a 70 μm strainer and cultured in complete Dulbecco’s Modified Eagle’s Medium (DMEM). A homogeneous culture of canine MSCs (dMSCs) was obtained after several passages following a previously described protocol [7]. dMSC and tumor cells (from surgery) were cultured in complete DMEM, supplemented with 10% heat-inactivated fetal bovine serum (FBS), 1% L-glutamine, streptomycin (100 mg/mL) and penicillin (100 U/mL), at 37 °C under humidified atmosphere with 5% CO_2_. Cells were fed every 2–3 days with complete DMEM. Additionally, all cells were routinely tested for *Mycoplasma* using the MycoAlert Mycoplasma Detection Kit (Lonza).

### 2.4. Preparation and Administration of dCelyvir 

Canine MSCs were transduced with an adenoviral vector expressing green fluorescent protein (GFP), and infected at a MOI (multiplicity of infection) of one for 1 h with the canine oncolytic adenovirus ICOCAV17 [24] (dCelyvir–GFP) [7]. Then, dCelyvir–GFP treatment was washed twice and resuspended in saline buffer ready to be administered using an IV catheter together with an anti-aggregate cell strainer. To prevent anaphylactic reactions, the dog was pretreated with metamizol (30 mg/kg/IV) and diphenhydramine (0.5 mg/kg/IV). dCelyvir–GFP was administrated at 0.5 × 10^6^ cells/kg of weight over 45 min. During the treatment, temperature, pulse and breathing rate of the canine patient was monitored every 10 min. 

### 2.5. Lung Carcinoma Sample Processing

The excised tumor was sliced, and samples were processed and stored under different conditions in order to be analyzed following diverse protocols. One part was fixed in 10% buffered formalin, and tumor specimens were prepared with hematoxylin and eosin stain for histological examination. The histopathological analysis of the lung mass was performed by a veterinary pathologist from the Pathology Service at the Complutense Veterinary Teaching Hospital (HCVC, UCM). The presence of a bronchial adenocarcinoma grade II with metastasis in the peribronchial lymph node was confirmed. One sample was stored with Trizol reagent at −80 °C; another sample was embedded in Tissue-Tek (OCT compound) and cryopreserved at −80 °C; and other samples were stored with phosphate-buffered saline (PBS) at 4 °C for performing cellular cultures and flow cytometry analysis.

### 2.6. Release of ICOCAV17 by the Tumor 

The cryopreserved tumor cells were thawed and were cultured in DMEM culture medium for 2 days. After that, culture supernatant was collected and divided into two samples: (1) DNA was extracted from fresh samples and (2) supernatant was used to infect DK28Cre cells. DK28Cre cells were seeded in a Nunc™ Lab-Tek™ Chamber Slide System (ThermoFisher Scientific, Waltham, MA, USA), and treated with the supernatant obtained from the primary tumor tissue culture. After 36 h, cells were fixed with ice-cold methanol and were washed twice with PBS 1% BSA. Cells were incubated overnight at 4 °C with the primary antibody anti-Ad5 (rabbit polyclonal to Adenovirus Type 5, Abcam, Cambridge, UK). After washing them with PBS 1% BSA, cells were incubated with Alexa488-conjugated secondary antibody (donkey anti-rabbit, Invitrogen, Waltham, MA, USA). After a further wash, preparations were mounted with ProLong™ Gold Antifade Mountant with DAPI (ThermoFisher Scientific, Waltham, MA, USA), and cell cultures were analyzed using the Leica DM4 B upright digital microscope (Leica Microsystems, Wetzlar, Germany).

### 2.7. DNA Extraction

A tumor sample (50 mg) was preserved at −80 °C with 1 mL of Trizol reagent until processed. The sample was mechanically homogenized and lysed for 5 min at 30 °C. DNA was extracted using a specific kit and following the manufacturer’s instructions. Peripheral blood from the patient was collected on days 2, 3, 7 and 14 after the treatment with dCelyvir–GFP. Additionally, as a control sample, peripheral blood of a healthy dog was collected and mixed with 10^8^ vp/mL of ICOCAV17. DNA was extracted from 1 mL of peripheral blood according to the manufacturer’s instructions of the QIAamp DNA Blood Mini Kit (Qiage, Hilden, Germany). DNA was extracted from the culture supernatant using the PureLink™ Viral RNA/DNA Mini Kit (Invitrogen, Carlsbad, CA, USA), following the manufacturer’s instructions. DNA quantification and purity analyses (A260/280 and A260/230) were carried out using the spectrophotometer Nanodrop (Thermo Scientific, Waltham, MA, USA) and ultimately DNA samples were preserved at −20 °C.

### 2.8. Quantitative Real-Time PCR 

To detect and quantify viral particles of ICOCAV17 in samples, DNA isolated from peripheral blood and the cell culture supernatant was analyzed by qPCR. DNA from the control sample (described above) was used to prepare serial dilutions from 10^7^ to 10^4^ vp/mL. These conditions were used as the standard curve in the qPCR. DNA samples were diluted to 50 ng/mL (except for the sample from the supernatant cell culture), and triplicates were analyzed by TaqMan system with a probe labeled with FAM and quenched by TAMRA. DNA and Premix Ex Taq (Clontech, Mountain View, CA, USA) were mixed with the forward primer (0.5 μmol/L) 5′-TGTGGGCCTGTGTGATTCCT-3′, the reverse primer (0.5 μmol/L) 5′-CCAGAATCAGCCTCAGTGCTC-3′ and 10 pmol of Taqman probe FAM-CTCGAATCAGTGTCAGGCTCCGCA-TAMRA. The qPCR protocol involved 40 cycles of a 10 min holding stage at 95 °C and 15 s cycles at 95 °C, followed by 1 min at 60 °C. The standard curve was used to determine the Ct number for each concentration of viral particles (10^7^ to 10^4^ vp/mL). The Ct number detected in each sample well was interpolated on the standard curve, and the mean of triplicates was calculated for each sample, to quantify the copy number of ICOCAV17. Quantitative real-time PCR was performed using the QuantStudio 3 Real-Time PCR System (Applied Biosystems, Waltham, MA, USA) and analyzed with QuantStudio 3 software (Applied Biosystems, Waltham, MA, USA).

### 2.9. Fluorescence Microscopy 

The cryopreserved tumor sample in OCT Tissue Teck and stored at −80 °C was sliced using a cryostat (Leica CM1520, Leica Microsystems, Wetzlar, Germany). Slices were stained with DAPI, and images were acquired by fluorescence microscopy (Leica DM4 B upright digital microscope, Leica Microsystems, Wetzlar, Germany). The tumor sample stored at 4 °C in PBS was disaggregated and digested by collagenase D during 45 min at 37 °C. The cellular suspension was filtered through a 70 μm strainer and cultured in complete DMEM. Cell culture images were taken 15 days after the IV administration of dCelyvir–GFP to the dog.

### 2.10. Flow Cytometry 

The tumor sample stored at 4 °C with PBS were disaggregated and digested by collagenase D for 45 min at 37 °C. The cellular suspension was filtered through a 70 μm strainer, resuspended in PBS and analyzed by flow cytometry to quantify the expression of GFP fluorescence. Data acquisition was performed with a MacsQuant10 flow cytometer (Miltenyi Biotec, Bergisch Gladbach, Germany), and data were analyzed using the MACSQuantify software (Miltenyi Biotec, Bergisch Gladbach, Germany). Every day, the laser settings of the instrument were calibrated routinely with MACSQuant^®^ Calibration Beads (Miltenyi Biotec, Bergisch Gladbach, Germany). The first gating strategy was removed doublets using forward scatter (FSC) and side scatter (SSC) parameters. Then, using FSC versus SSC gating, cellular debris was eliminated to identify the GFP-positive cells.

### 2.11. Statistical Analysis 

Data were analyzed using GraphPad Prism software (GraphPad Software, La Jolla, CA, USA). Graphical representation of data involves mean + SD.

## 3. Results

**Figure 1:**
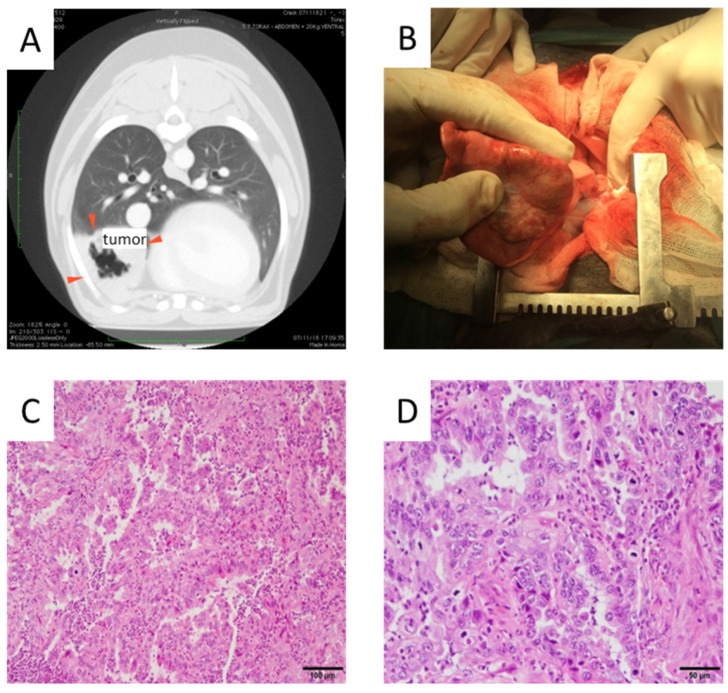
Tumor images. (**A**) Computerized tomography image prior to surgery indicating tumor location (red arrows). (**B**) Tumor mass observed during the lung lobectomy. (**C**,**D**) Hematoxylin and eosin-stained pulmonary adenocarcinoma is shown. Papillary predominance with a mild to moderate fibrovascular core lined by cuboidal to columnar cells is shown. Neoplastic cells with moderate eosinophilic cytoplasm and round to ovoid nuclei with granular or vesicular chromatin and 1–2 magenta nucleoli are shown. Scale bars: 100 µm (**C**) and 50 µm (**D**).

To confirm the tumor-homing capacity of infected dMSCs after an IV administration of dCelyvir–GFP treatment, an in vivo study using a canine patient with a spontaneous lung carcinoma was performed. The patient presented with a right intrathoracic mass diagnosed by thoracic radiology procedures. An ultrasound-guided fine-needle aspiration of the mass confirmed a malignant epithelial tumor, and an ultrasound examination of the abdomen was normal. Since the thoracic computerized tomography showed the presence of a tumor confined into the right middle lobe of the lung and an enlarged tracheobronchial lymph node, lung lobectomy and removal of the affected lymph node were indicated (Figure 1A,B). The tumor biopsy showed moderate pleomorphism and a high mitotic rate (Figure 1). Microscopy of the tissue showed numerous neutrophils in the alveolar lumen and in the tumoral stroma (Figure 1C), some scattered necrotic cells and some neutrophils in the alveolar lumen and in the tumoral stroma (Figure 1D). The histopathological analysis of the lung mass confirmed the presence of a bronchial adenocarcinoma grade II with metastasis to the peribronchial lymph node.

**Figure 2:**
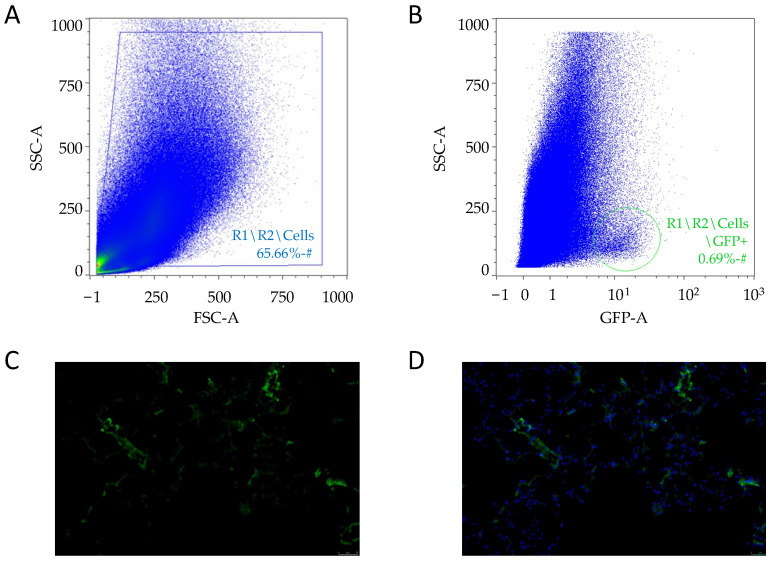
Tumor cells analysis. (**A**,**B**) Plots showing the events acquired by flow cytometry after excluding the cell doublets. The cellular debris excluded (**A**) and GFP-positive cells (circled) after the strategy gating (**B**). (**C**,**D**) Sections of tumor biopsies from lung carcinoma embedded in OCT. Representative images after 48 h of treatment with dCelyvir–GFP. GFP fluorescence in cells (**C** and **D**; green) and nuclear staining with DAPI (**D**; blue). Scale bars, 50 µm.

dCelyvir was administered 48 h prior to the surgery. A GFP-positive cell population (0.69%) was detected by flow cytometry when the excised canine lung carcinoma tissue was analyzed (Figure 2A,B), confirming the presence of dMSC inside the tumor. GFP-positive cells were detected also by fluorescence microscopy in cryopreserved tumor biopsies (Figure 2C,D), which confirmed the presence of the cellular treatment inside the carcinoma tumor sample.

**Figure 3:**
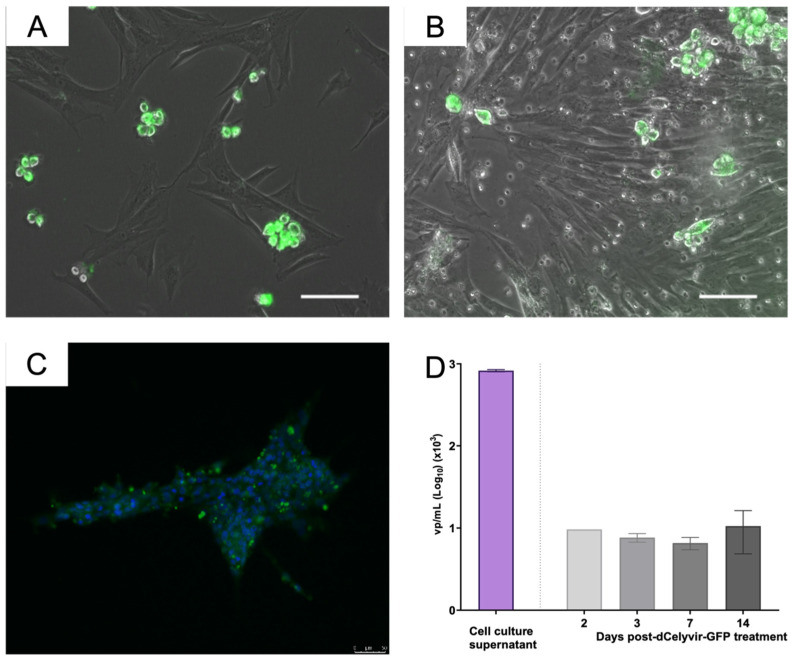
Tumor cell culture and ICOCAV quantification. (**A**,**B**) Cell culture from lung carcinoma. Representative images of cellular culture at days 14 (**A**) and 15 (**B**) after dCelyvir treatment show adenovirus detection (green fluorescence) in some cells. GFP-positive cells are grouped and present round morphology. (**C**) DK28Cre cell culture treated with supernatant released from cell culture from tumoral lung biopsy. Representative immunofluorescence image after 36 h of treatment shows adenovirus detection (green fluorescence) in cells. Nuclear staining with DAPI (blue). Scale bars, 50 µm (**A**–**C**). (**D**) Adenoviral DNA quantification by qPCR. Quantification of viral particles (vp) in supernatant released from cell culture established from the biopsy lung tumor and vp in peripheral blood at different time points after dCelyvir–GFP treatment are shown. Data were analyzed using GraphPad Prism software (GraphPad Software). Mean values + SD are shown.

Furthermore, the presence of GFP fluorescence expression inside the tumor was confirmed by evaluating the cell cultures obtained from lung tissue. Green, fluorescent cells were detected by microscopy in cell cultures from the lung carcinoma samples 14 and 15 days post-administration (Figure 3A,B). These results indicate that the inoculated MSCs reached the lung carcinoma tissue. The presence of ICOCAV17 was also analyzed in cell culture established from the lung biopsy. The supernatant released by these cells was analyzed by qPCR to quantify the ICOCAV17 (Figure 3D). The quantification of the viral particles showed a high genome copy number of ICOCAV17. Furthermore, the viral particles detected in the supernatant were infectious, since they infected DK28Cre cell culture, as demonstrated by detecting particles in the cell culture by immunofluorescence after 36 h (Figure 3C,D). These results indicate that dCelyvir reached the tumor and released functional oncolytic adenovirus.

To determine whether the ICOCAV17 was present in the peripheral blood from the canine patient, different samples were evaluated over time after treatment (at days 2, 3, 7 and 14). Quantification of the viral particles showed that adenoviral DNA was present in peripheral blood for up to 14 days after IV administration of dCelyvir–GFP (Figure 3E). In addition to efficiently reaching the lung tumor tissue, the presence of adenoviral DNA in peripheral blood suggests that dCelyvir treatment allowed the replication and release of virus from tumor tissue into the blood.

## 4. Discussion

Since the approval of the oncolytic virus therapy Imlygic as an anticancer therapy by the FDA and EMA, the use of oncolytic viruses has increasingly become a promising cancer treatment option [25,26]. In veterinary medicine, oncolytic viruses have been used as experimental treatments for oncologic patients [6,24,27]. Various in vivo trials and in vitro experiments with the measles virus [28], ICOCAV [7], vaccinia virus [29] and canine distemper virus have reportedly confirmed the efficacy of these treatments [30,31]. Even though the effectiveness of the treatment is directly related to the selective replication of the virus in the tumor cells, the administration route of the virus remains a key point for improvement [1,14].

The administration of the OVs intravenously must overcome many physiological barriers, which effectively reduce the OVs dose, and therefore, their efficacy is decreased [18]. Due to the tumor tropism of MSCs, their use as OVs vehicles has been proposed [7,17,18]. The homing capacity of MSCs infected with OAd has been discussed in several murine models, such as the MDA-MB-231 murine pulmonary breast metastasis model [21] or the C57BL/6 murine lung carcinoma model [17], but a study with the involvement of human or canine clinical patients has not yet taken place [7,32].

In a reported veterinary clinical trial with dCelyvir, the highest amount of adenoviral DNA in blood samples was detected 72 h after the first infusion [7]. In our patient it was identified as early as 48 h after administrating the therapy. It could be possible that the used therapy reached other organs, which decreased the percentage of GFP-positive cells that reached the tumor [33]. However, even considering the cells that reached other organs, the dose that presumably reached the tumors in a previous study was sufficient to have a clinical effect, judging from the responses seen in the 74% of the treated dogs [7]. Our canine patient suffered a relapse after the surgery, which is suggestive of a possibly preexisting micrometastasis before the dCelyvir was administered. Previous studies have reported results where the oncolytic virus has been detected in metastases in dogs treated with dCelyvir [7]. In addition, the treatment does appear to influence metastases, since at least one of the treated dogs in previous studies had a complete remission (CR) of lung metastases after systemic treatment. It should be noted that the CR was obtained with weekly repeated doses of the treatment; however, in the present case report, a single treatment dose was administered, since the standard of care was surgery. It could be suggested that just one dose might not be enough to have any efficacious results on metastases.

## 5. Conclusions

This study provides insights into the understanding of the dCelyvir response mechanism, and shows for the first time the presence of MSCs infected with an oncolytic adenovirus in the tumor tissue of a patient. This confirms the tumor-homing capacity of Celyvir therapy in a clinical setting. It also suggests that the effect described in previous publications is not only due to the activation of the immune system, but also because the virus plays an important role within the tumor.

## Data Availability

Not applicable.
